# Transcriptome analysis reveals nutrient deprivation reduces nitrate content in lettuce (*Lactuca sativa* var. *ramosa* Hort.) and enhances nitrogen metabolism

**DOI:** 10.3389/fpls.2025.1585955

**Published:** 2025-05-19

**Authors:** Yingying Zhang, Xuena Liu, Shikai La, Mingjiao Wang, Xu Hu, Ainong Shi, Jinghua Guo, Lingdi Dong

**Affiliations:** ^1^ Institute of Cash Crops, Hebei Academy of Agriculture and Forestry Sciences, Shijiazhuang, China; ^2^ Institute of Coastal Agriculture, Hebei Academy of Agriculture and Forestry Sciences, Tangshan, China; ^3^ Department of Horticulture, University of Arkansas, Fayetteville, AR, United States

**Keywords:** lettuce, nutrient deprivation, nitrate, N metabolism, transcriptome analysis

## Abstract

**Introduction:**

Excessive nitrate accumulation in leafy vegetables poses health risks and indicates inefficient nitrogen use in conventional agriculture. While nitrogen metabolism has been extensively studied, the molecular mechanisms linking nutrient deprivation to nitrate reduction in vegetables remain unclear.

**Methods:**

In this study, we investigated these mechanisms in lettuce (*Lactuca sativa* var. *ramosa* Hort.) by analyzing nitrate content and gene expression in leaves and roots under nutrient deprivation.

**Results:**

After five days of treatment, nitrate content decreased by 57.49% in leaves and 50.77% in roots. Transcriptome sequencing identified 323 and 3,494 differentially expressed genes (DEGs) in leaves and roots, respectively, with 78 shared DEGs. KEGG enrichment analysis revealed involvement of DEGs in pyrimidine metabolism, base excision repair, hormone signaling, terpenoid biosynthesis, and triglyceride metabolism, indicating cross-talk between nitrate regulation and stress responses. Nitrate transporter genes *NRT2.4* and *NRT2.5* were upregulated in roots, while *NRT1* was induced in leaves, suggesting enhanced nitrate redistribution. Additionally, antioxidant genes such as *POD*, *LOX*, and cytochrome P450 were upregulated in roots, whereas *SODC* was downregulated in both tissues. These results suggest that lettuce responds to nutrient deprivation by activating nitrate transport and antioxidant pathways to reduce nitrate levels and enhance nitrogen use efficiency.

**Discussion:**

This study provides a foundation for optimizing pre-harvest strategies to improve lettuce quality and identifies candidate genes (e.g., *NRT2.5*, *LOX*) for breeding low-nitrate varieties suited for nitrogen-limited environments.

## Introduction

Nitrogen is indispensable for the synthesis of amino acids, nucleotides, and chlorophyll (Tatiana et al., 2011; Meng et al., 2015; Xiong et al., 2018; Reddy et al., 2020). Nitrate, the primary form of nitrogen absorbed by plants, is taken up through roots and assimilated into organic compounds (Vandna et al., 2024). The absorption and metabolism of nitrate are influenced by multiple factors, including nutrient availability, environmental conditions, and the physiological state of the plant (Yuan et al., 2021; Rui et al., 2023; Vandna et al., 2024). Nutrient availability in the soil is not only affected by nitrogen fertilizer application strategies but also by factors such as soil pH, ion competition, and microbial activity. Environmental conditions, such as photoperiod, can further regulate nutrient uptake through hormone signaling pathways (Noorden et al., 2016; Dai et al., 2024). From a physiological perspective, nitrate demand varies across developmental stages—being higher during vegetative growth—and is further influenced by abiotic stresses such as drought or nitrogen deficiency, which can activate nitrogen utilization pathways to maintain homeostasis, for example, through glutathione biosynthesis or reduced amino acid synthesis (Li et al., 2023). However, nitrogen regulation and utilization are complex, and plant responses to nitrogen deficiency can vary across species and conditions (Yin et al., 2019). These complex regulatory networks form the molecular basis for precision agriculture practices aimed at improving nitrogen use efficiency. Future research must therefore integrate multi-omics technologies to elucidate the synergistic interactions among environmental signals, gene expression, and metabolism.

Lettuce (*Lactuca sativa* var. *ramosa*) is a widely cultivated leafy vegetable with high nutritional value (Kim et al., 2016). However, its classification as a high-nitrogen-demand crop presents a critical challenge: while sufficient nitrogen promotes biomass accumulation, excessive nitrate accumulation in edible tissues poses food safety risks. Epidemiological data estimate that vegetables contribute approximately 80% of total dietary nitrate intake in humans (Liu et al., 2011a; Li et al., 2013), making nitrate management in crops like lettuce a priority for sustainable agriculture. Previous research indicates that nutrient deprivation can trigger nitrate remobilization through autophagy-mediated protein degradation and upregulation of vacuolar nitrate efflux transporters (Yuan et al., 2007; Rengel et al., 2022). However, tissue-specific responses—especially in leafy vegetables where leaf nitrate content directly impacts food safety—remain poorly understood. Lettuce is highly responsive to nutrient availability, and nutrient deprivation not only affects its growth and productivity but also significantly influences nitrate content, with direct implications for human health (Zhao et al., 2024). Thus, studying nutrient deprivation in lettuce offers a unique opportunity to deepen our understanding of nitrate metabolism and provide foundational knowledge for high-yield, high-quality hydroponic cultivation. Despite its importance, the mechanisms by which nutrient deprivation influences nitrate metabolism in lettuce are still insufficiently explored.

Transcriptome analysis offers powerful insights into gene expression changes under specific physiological conditions. Transcription factors (TFs) play crucial roles in modulating gene expression and regulating metabolic pathways (Fan et al., 2023). Advances in transcriptomics have enabled the identification of key genes and TFs involved in metabolite biosynthesis and stress responses (Huang et al., 2023). For example, *Dof1.7* and *NIGT1* TFs mediate complex regulation of *NRT2* gene expression under nitrogen deficiency (Zhuo et al., 2024), while transcriptomic studies in wheat have revealed calcium-mediated signaling pathways involved in nitrogen stress tolerance (Yan et al., 2021). These findings highlight the potential of tissue-specific transcriptomics to uncover both conserved and species-specific regulatory mechanisms. Nonetheless, current knowledge is largely derived from model species and cereals, with limited data available for fast-growing leafy vegetables, in which nitrate accumulation has direct consequences for consumer health and marketability.

Optimizing nutrient management can effectively reduce nitrate accumulation in crops. For instance, Song et al. (2023) demonstrated that nutrient deprivation five days prior to harvest significantly lowered nitrate content in *Brassica rapa* ssp. *chinensis*. This study provides a useful reference for our research. Here, we examine the impact of nutrient deprivation on nitrate content in lettuce under standard and reduced nutrient conditions. Using transcriptome sequencing, we analyze gene expression patterns in lettuce roots and leaves. This study establishes a theoretical framework for understanding the molecular response of lettuce to nutrient deprivation and provides practical guidance for optimizing nitrogen management in lettuce production systems.

## Materials and methods

### Plant materials and experimental conditions

The lettuce variety *Lactuca sativa* (Dasusheng, Beijing Fengming Yashi Technology Development Co., Ltd., Beijing, China) was selected for this study. Seeds were sown in cavity trays filled with a substrate mixture of cottonseed husk and vermiculite (2:1 ratio).

Lettuce was grown under hydroponic conditions, following the methodology outlined in our previous study (Song et al., 2023). After 25 days of growth in a complete nutrient solution, seedlings exhibiting consistent growth were selected and transferred to two identical hydroponic racks for cultivation. One rack was subjected to nutrient deprivation, where the nutrient solution was completely replaced with water, thereby completely eliminating the supply of essential nutrients to the plants. The other hydroponic rack maintained unchanged culture conditions and served as the control group (CK). A harvest of the control group plants was performed just before the onset of nutrient deprivation, corresponding to day 0 (D0) of the nutrient deprivation experiment.

### Determination of biomass and nitrate content

On the day of nutrient deprivation application (D0), control plants were harvested in three replicates, each consisting of four plants, to ensure an adequate sample size for subsequent analysis. Samples were collected daily thereafter. The fresh weight of each tissue sample was recorded, and each sample was then divided into two homogeneous batches. One batch was stored at -80°C for molecular analysis, while the other was used for nitrate content determination. Nitrate content was measured using salicylic acid-sulfuric acid colorimetry, as described by Cao et al. (2007). Data were analyzed using one-way ANOVA followed by Tukey’s test (SPSS v27.0).

### RNA extraction and Illumina sequencing

On the 5th day, leaves (CL, TL) and roots (CR, TR) from both the control group and the nutrient deprivation treatment were immediately frozen in liquid nitrogen and stored at -80°C. The tissue samples were then sent to BMK for transcriptome sequencing.

Total RNA was extracted from the leaves and roots of lettuce under both conditions (CL/CR for normal nutrient supply and TL/TR for nutrient deprivation) using a NanoDrop 2000 (Thermo Fisher Scientific, Wilmington, DE). Twelve libraries representing four sample groups (three replicates per group) were constructed for transcriptome sequencing. RNA quality, quantification, and sequencing were conducted with the assistance of BMKCloud (www.biocloud.net). Following cluster generation, the library preparations were sequenced on an Illumina platform, and paired-end reads were produced. The transcriptomic samples and their corresponding experimental groupings are detailed in **Table 1**.

**Table 1:** Group of transcriptomic samples.

Position	Treatment	
	Normal nutrient supply	Nutrient deprivation
Leaf	CL (S391,S392,S393)	TL (S361,S362,S363)
Root	CR (S397,S398,S399)	TR (S367,S368,S369)

### Screening and functional analysis of DEGs

The sequencing data (clean data) were aligned using HISAT2 (v2.2.1) software, and the reads were spliced and assembled with StringTie to obtain reference sequences for subsequent analysis. High-quality reads were mapped to the spliced transcriptome to calculate gene expression levels. DEGs were identified using a fold change ≥ 1.5 and a P-value ≤ 0.01 as the screening criteria. Gene Ontology (GO) and KEGG enrichment analyses were then performed on the identified DEGs to explore their functional roles.

### Real-time PCR

Quantitative reverse transcription PCR (qRT-PCR) was performed to validate the RNA-seq results. RNA samples were reverse-transcribed using the Evo M-MLV RT Mix Kit (Accurate Biology). Primers for the analysis were designed with Primer 5.0 software (PREMIER Biosoft) (**Table 2**). The expression of the *Lactuca sativa* actin gene (LSAT_V11C800436410) was used as internal control. SG Green qPCR Mix (Bysbio) was used for real-time PCR reactions. The Ct values of DEGs and the internal reference gene were measured, and each gene was tested in triplicate. The 2^-ΔΔCt^ method was employed to calculate the relative expression levels of the genes.

**Table 2:** DEGs and their primer sequences in qRT-PCR.

Gene ID	Name of gene	Gene annotation	Primer sequence(5’-3’)
Lsat_1_v5_gn_2_94701	*GDPD2*	Glycerophosphodiester phosphodiesterase	F: ATTCAACCTGCCACCCTAC R: TCAACAACGCTGCCAAACA
Lsat_1_v5_gn_4_41841	*COG4*	Conserved oligomeric Golgi complex subunit	F: TCTGATGAAACGGAGGACA R: CTTTGAGTCGGCTGCTAAT
Lsat_1_v5_gn_8_70441	*COPT5*	Copper transporter	F: CAGCCCAACGACTATTACCA R: TGCGATTCAAGCTCCTTTC
Lsat_1_v5_gn_4_56181	*ZIP2*	Zinc transporter	F: GCCAGCACAAGCCAACATA R: GACGCCCAGAAACCCAACC
Lsat_1_v5_gn_3_21780	*RAP2-3*	Ethylene-responsive transcription factor	F: TCACCACCGAGTCCACCAA R: ACGATGAAGCAGCCAAACG
Lsat_1_v5_gn_7_106541	*CAB13*	Chlorophyll a-b binding protein 13	F: TGGACAAGGTTCGGGTTTC R: GCATTCGGATGTATCACGC
Lsat_1_v5_gn_9_105960	*ACO3*	1-aminocyclopropane-1-carboxylate oxidase 3	F: CAACAGGACCCAAACTCAC R: AAAGAACAATCGTACCCAAAG
Lsat_1_v5_gn_6_43340	*SODCP*	Superoxide dismutase [Cu-Zn]	F: TGAAATGTGGTCCTGTTGAT R: GTGTCGAAGGCGTTGTTAC
Lsat_1_v5_gn_0_680	*PETE*	Plastocyanin	F: GGAACCTCGTCTTCATCAAAC R: CCACCACTGCTACCGTCAA
Lsat_1_v5_gn_2_131181	*FRO4*	Ferric reduction oxidase	F: TGAGTGAAAGCCCAGTAGA R: CAAGTTCCGTAGTGTATCGT
Lsat_1_v5_gn_5_95460	*EBF2*	EIN3-binding F-box protein	F: GAAGGCAAGTTCCATAACG R: GACGGGTTTCTCACAAGGT
Lsat_1_v5_gn_4_77480	*NAR2.1*	High-affinity nitrate transporter-activating protein	F: TTATCAAGGGCGTCATCAG R: TGCTCACAATGTCCACCTC
Lsat_1_v5_gn_3_45820	*DIR4*	Dirigent protein	F: GTCCCACAGTGAGCCAGTC R: CATCCTTTCAGGCAACAATC
Lsat_1_v5_gn_8_137141	*FLA9*	Fasciclin-like arabinogalactan protein	F: CCATCTTCCGCACCACTCG R: ATTGAGACAGCAGTTTCCGTTTG
Lsat_1_v5_gn_2_104741	*CCH*	Copper transport protein	F: GTGCTCCAGATCAATGTCAAA R: CAGACCGTCGTTCTCAAGG
Lsat_1_v5_gn_2_108920	*PIP2-4*	Aquaporin	F: TTGAGACTTGTAGCCGATGAC R: GATTACGAAGACCCTCCACC
Lsat_1_v5_gn_9_43700	*RAP2-4*	Ethylene-responsive transcription factor	F: CCGCCGGATACCTCACTCT R: AACTTTCCTCACCTGCGTCAT
Lsat_1_v5_gn_8_78661	*TIP1-3*	Aquaporin	F: AAATGGGCCTAGCCAGTAA R: TGCGGTGGTGTTTGAGATA
	*LsACT*		F: ATCAGGGAGTCTGTGAGGT R: TCTATGCCAGTGGTCGTAC

## Results

### Nutrient deprivation triggers nitrate content reduction

**Figure 1:**
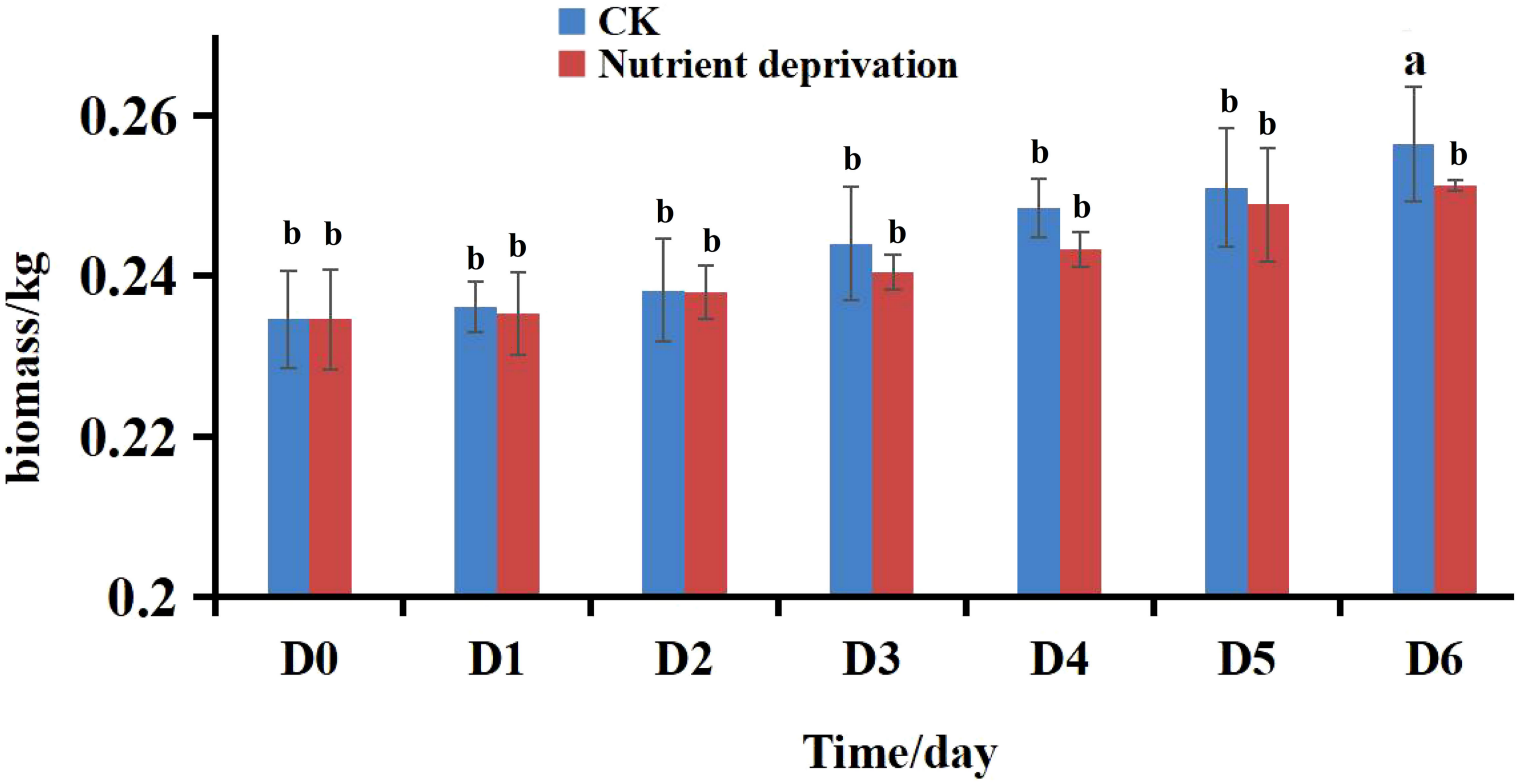
Biomass of lettuce under different conditions. Different lowercase letters in the same day indicate significant differences among different treatments at 0.05 level.

**Figure 2:**
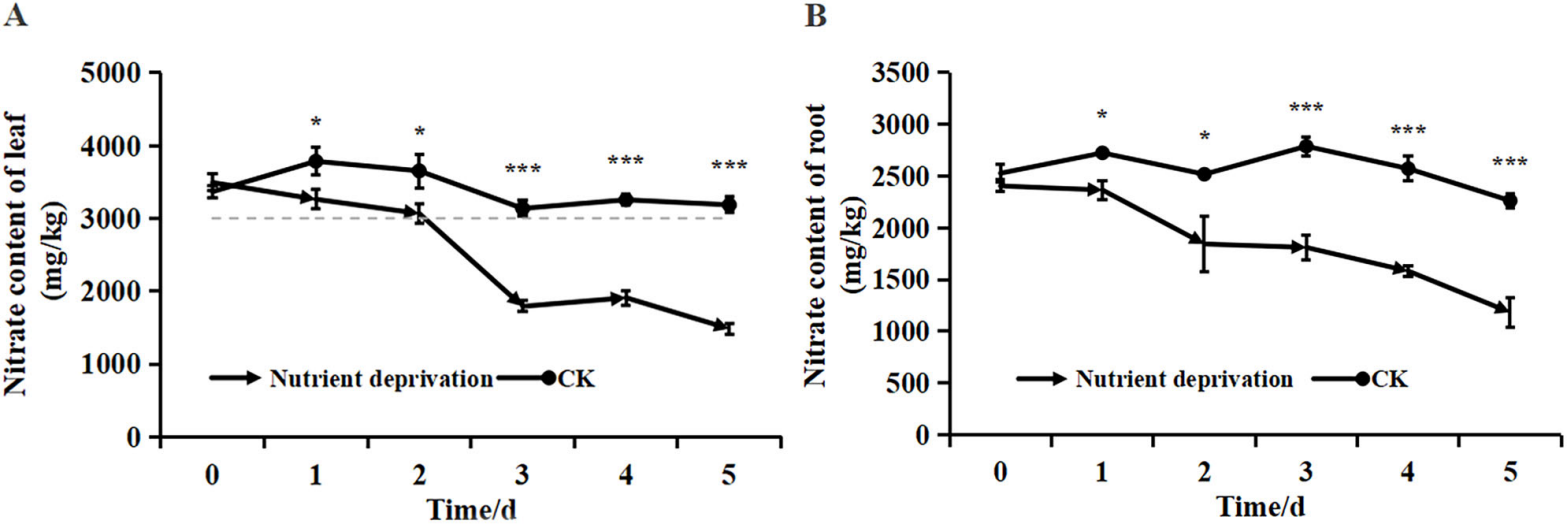
Nitrate content of lettuce **(A)** leaf; **(B)** root. Data represent means ± standard deviation, and asterisks indicate significant differences (*p<0.05, ***p<0.001).

The biomass of lettuce remained unaffected during the first five days of nutrient deprivation (**Figure 1**). While the nitrate content in both the leaves and roots of lettuce exhibited a rapid decline. The most substantial reduction in nitrate content occurred on day 3 in the leaves and day 2 in the roots. Overall, nitrate levels decreased by 57.49% in the leaves and 50.77% in the roots. By the end of the treatment, nitrate concentrations remained within the prescribed limit for green, pollution-free vegetables (less than 3000 mg/kg) (**Figure 2**). It shows that short-term nutrient deprivation can be used as an effective nitrate reduction strategy.

### Transcriptome sequencing and alignment

Transcriptome sequencing of lettuce roots and leaves generated a total of 80.86 Gb of clean data after quality control (**Table 3**). The sequencing quality was high, with the percentage of Q30 bases (sequencing error rate ≤ 0.1%) in all samples exceeding 91.61%. The GC content of the samples ranged from 44.50% to 45.55%. The comparison efficiency between the reads of each sample and the reference genome ranged from 83.17% to 96.14%, indicating strong alignment and providing reliable data for further analysis.

**Table 3:** Transcriptome data statistics of lettuce samples.

Condition	Name	Clean reads	Reference genome mapping/%	Q30/%	Content/%	Total reads
CK	S391	17096427	94.11%	91.61%	45.13%	34192854
	S392	18358406	94.57%	92.27%	45.25%	36716812
	S393	23886176	96.14%	92.64%	45.55%	47772352
	S397	21915781	90.73%	92.09%	44.74%	43831562
	S398	32008934	91.30%	91.86%	44.77%	64017868
	S399	19632364	83.17%	93.81%	45.21%	39264728
T	S361	20756800	94.89%	93.79%	45.46%	41513600
	S362	25383548	96.14%	94.37%	45.46%	50767096
	S363	22785258	95.37%	93.98%	44.65%	45570516
	S367	23190196	92.67%	92.88%	44.60%	46380392
	S368	20923665	93.87%	92.54%	44.50%	41847330
	S369	23964751	92.58%	92.87%	44.59%	47929502

**Figure 3:**
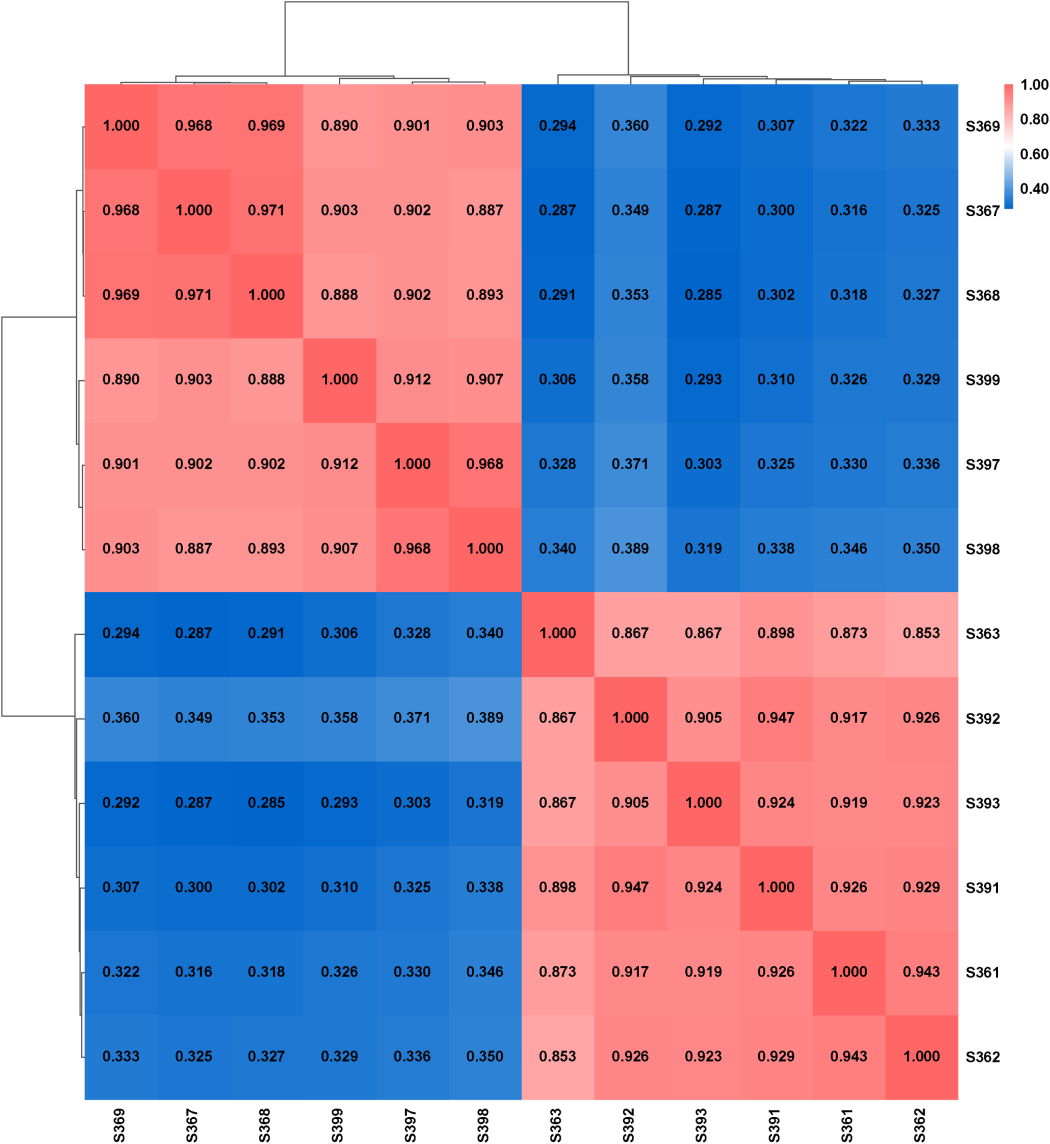
Correlation heatmap of gene expression.

Spearman’s correlation coefficient (r) was used to assess the correlation between biological replicates based on the quantitative FPKM (Fragments Per Kilobase of exon model per Million mapped reads) results. The closer the correlation coefficient (r²) is to 1, the stronger the correlation between the two replicate samples. A heat map (**Figure 3**) was generated to visualize gene expression correlations across the 12 samples. As shown in the figure, the correlation between the three biological replicates for both the leaf and root samples remained consistently above 0.853. This indicates a strong correlation among the replicates, which supports the reliability of the experimental results and the appropriateness of the sample selection.

### DEGs in leaves and roots of lettuce under nutrient deprivation

**Figure 4:**
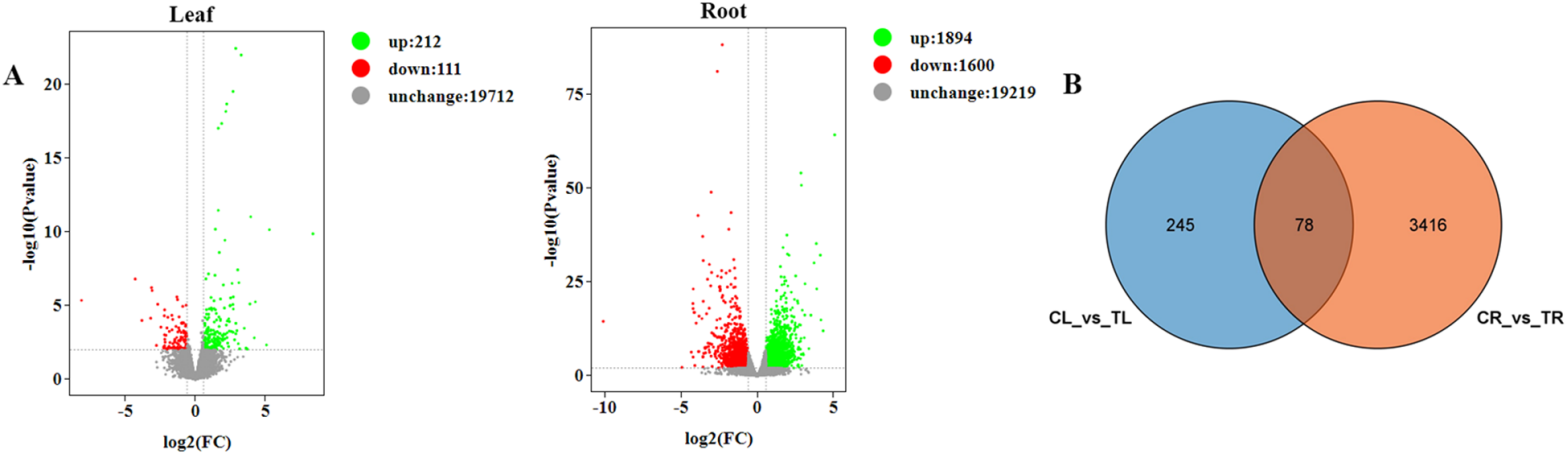
DEGs analysis of leaves and roots under nutrient deprivation conditions. **(A)** Volcano plot of DEGs of different positions (roots, leaves) under nutrient deprivation. **(B)** Venn diagram displaying the DEGs in leaf (CL_vs_TL) and root (CR_vs_TR) under nutrient deprivation.

DEGs between nutrient deprivation and control samples were screened and identified using the DESeq2_edgeR. DEGs were selected based on a fold change (FC) ≥ 1.5 and a significant difference (P-value ≤ 0.01). To identify genes associated with nitrate accumulation and explore the relationship between DEGs in lettuce roots and leaves under nutrient deprivation conditions, expression levels were analyzed and compared with the control group. Differential expression analysis revealed 323 DEGs in the leaves, consisting of 212 upregulated genes and 111 downregulated genes. In the roots, 3494 DEGs were identified, with 1894 upregulated genes and 1600 downregulated genes (**Figure 4A**). Notably, 78 DEGs were found in both the leaves and roots (**Figure 4B**). The number of DEGs in roots was greater than those in leaves, indicating that the roots undergo more significant differential expression, which might be because roots were the first to respond to stress stimuli.

**Figure 5:**
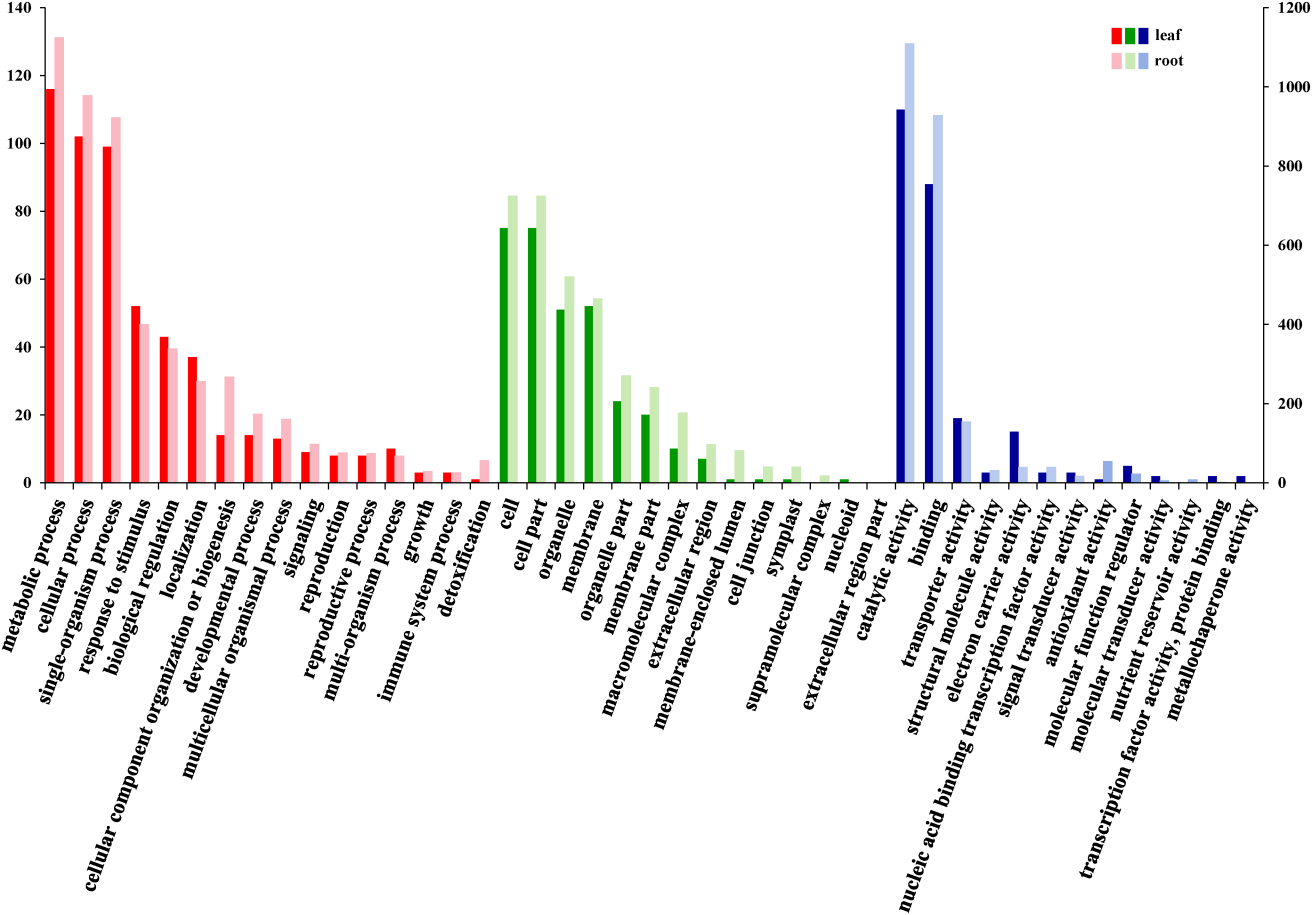
GO analysis of DEGs (red) biological process; (green) cell component; (blue) molecular function.

191 DEGs in the leaves and 1955 DEGs in the roots were enriched in three main GO categories: biological process, cellular component, and molecular function (**Figure 5**), which suggest that the DEGs play roles in essential cellular processes, highlighting their potential involvement in nitrate accumulation and the plant’s response to nutrient deprivation.

**Figure 6:**
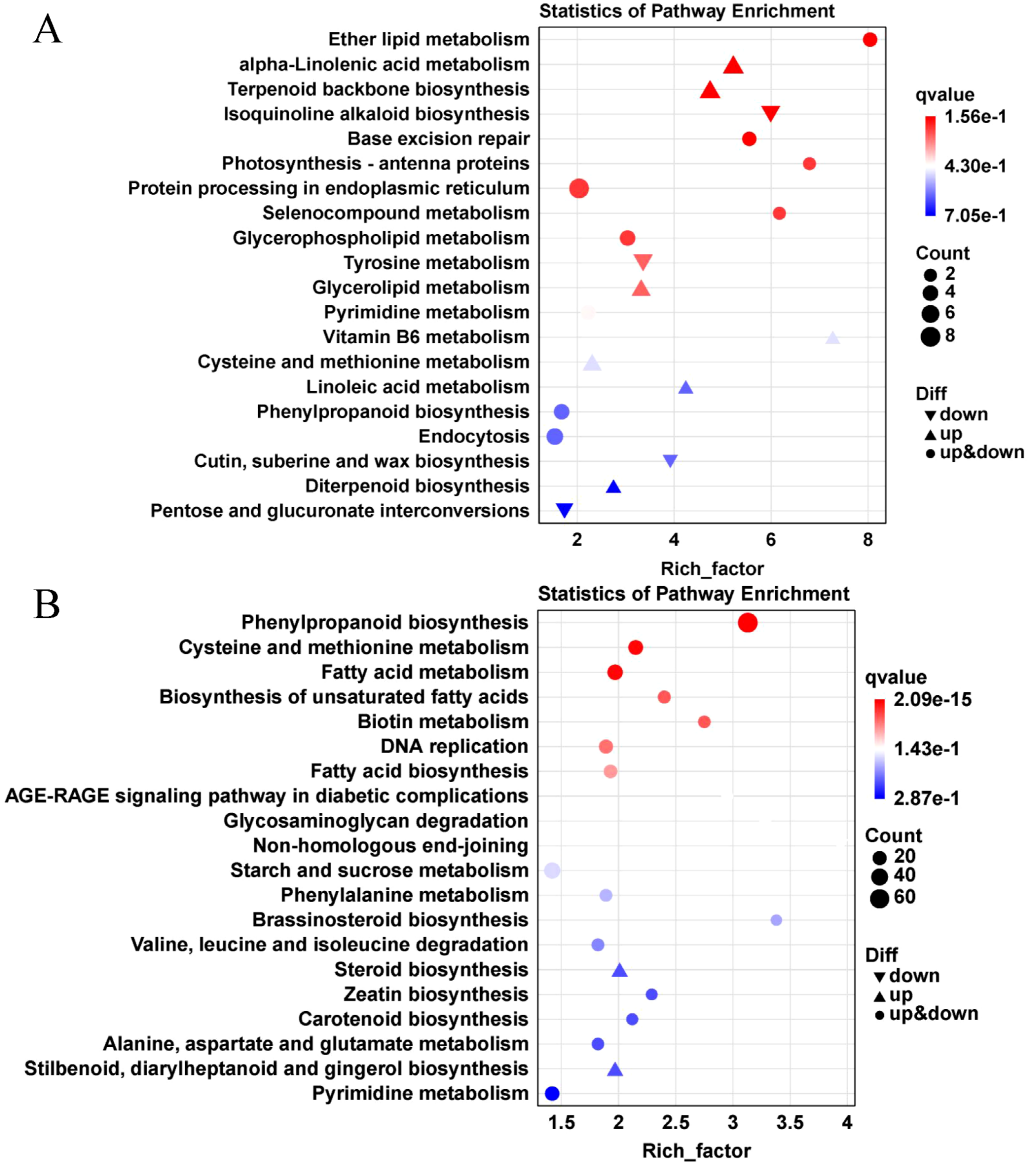
The top 20 KEGG pathway enrichment analysis of DEGs after nutrient deprivation. The picture shows the top 20 KEGG pathway enrichment bubble of DEGs in leaves **(A)** and roots **(B)** after nutrient deprivation; The X-axis represents the enrichment factor value (Rich Factor). The larger the data, the more obvious the enrichment results. The Y-axis represents the path name. The size of the dots represents the number of DEGs. The depth of the dots indicates the Q-value, the smaller the value, the more significant the enrichment results.

Low nitrogen stress impacts secondary metabolic pathways in plants, aiding in stress mitigation and damage repair. These pathways include phenylpropanoid metabolism, with sub-pathways like lignin and flavonoid biosynthesis. Studies have shown that regulatory factors influence nitrogen metabolism by modulating target genes at the transcriptional level (Liu et al., 2024b). To further explore the metabolic responses of lettuce to nutrient deprivation, the top 20 most significantly enriched pathways in DEGs from both leaves and roots were analyzed (**Figure 6**).

In leaves, DEGs were primarily enriched in pathways such as protein processing in the endoplasmic reticulum (duress-based repair), and plant hormone signal transduction (growth regulation). In roots, DEGs were enriched in phenylpropanoid biosynthesis (antioxidant), starch and sucrose metabolism (carbon-nitrogen balance), and amino acid biosynthesis (nitrogen reuse). These pathways are involved in antioxidant defense and growth regulation, indicating the plant’s adaptive mechanisms to nutrient deprivation stress.

**Figure 7:**
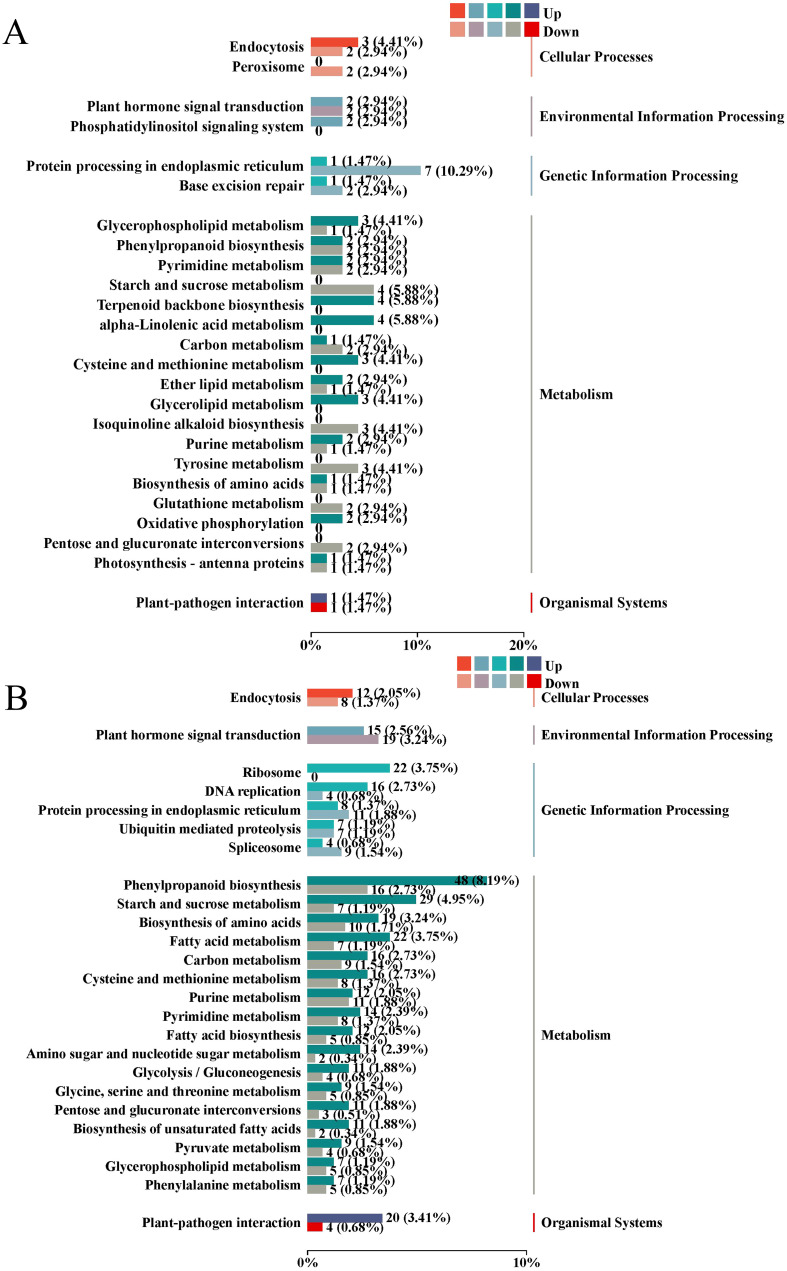
KEGG classification of DEGs **(A)** leaf; **(B)** root.

Functional enrichment analysis of upregulated and downregulated DEGs in lettuce leaves and roots after nutrient deprivation was conducted using the KEGG database. The results (**Figure 7**) revealed the following:

- Leaves: The upregulated DEGs were significantly enriched in α-linolenic acid metabolism, terpenoid backbone biosynthesis, cysteine and methionine metabolism, endocytosis, glycerolipid metabolism, and glycerophospholipid metabolism. The downregulated DEGs were concentrated in protein processing in the endoplasmic reticulum, starch and sucrose metabolism, isoquinoline alkaloid biosynthesis, and tyrosine metabolism.
- Roots: The upregulated DEGs were mainly enriched in phenylpropanoid biosynthesis, starch and sucrose metabolism, ribosome function, fatty acid metabolism, plant-pathogen interaction, amino acid biosynthesis, carbon metabolism, cysteine and methionine metabolism, and DNA replication. The downregulated DEGs were primarily involved in plant hormone signal transduction, purine metabolism, protein processing in the endoplasmic reticulum, spliceosome function, α-linolenic acid metabolism, and glutathione metabolism.

These findings highlight significant differences in the nitrogen response genes between the leaves and roots of lettuce, with distinct enrichment in pathways related to carbon metabolism (such as starch and sucrose metabolism) and nitrogen metabolism (such as amino acid metabolism) under nutrient deprivation conditions.

### Analysis of DEGs associated with nutrient deprivation

To explore the molecular mechanisms underlying lettuce’s response to nutrient deprivation stress, we further screened key genes among the DEGs. A total of 130 TFs, classified into 23 families, were identified in the root transcripts (TR). The AR2/ERF family was the most abundant, with 35 genes (26.92%), followed by WRKY (25 genes, 19.23%), bHLH (16 genes, 12.31%), MYB (15 genes, 11.54%), and NAC (5 genes, 3.85%). In leaf tissues, 11 TFs were identified in response to nutrient deprivation stress, classified into 5 TF families, including ERF, bHLH, and MYB. Notably, 3 MYB family TFs (*MYB3, MYB108, APL*) were upregulated. A partial list of TFs involved in NO_3_^-^ response is provided in **Table 4**. These TFs likely play a critical role in lettuce’s adaptation to nutrient deprivation stress.

**Table 4:** Partial TFs involved/potentially involved in NO_3_^-^ response.

Position	TF-Family	TF	Log_2_FC (T/CK)
LEAF	AR2/ERF	*ANT*	3.07
		*ERF4*	0.73
		*RAP2-3*	0.69
	MYB	*MYB3*	2.27
		*MYB108*	1.70
	bHLH	*bHLH35*	2.71
		*bHLH48*	-0.64
		*bHLH137*	-1.60
ROOT	bZIP	*RF2B_ORYSJ*	1.41
	MADS-box	*MADS6_ORYSJ*	-1.58
	NAC	*NAC25*	2.27
		*NAC29*	-0.82
		*NAM-B1*	1.86
	AR2/ERF	*ERF021*	2.65
		*ERF2*	1.85
		*RAP2-11*	-1.18
	WRKY	*WRKY60*	1.44
		*WRKY70*	2.09
		*WRKY18*	2.04
		*WRKY48*	-1.52
		*WRKY23*	-1.63

Log_2_FC means log_2_FoldChange (T/CK).

### Essential DEGs in nitrate transport and assimilation

To understand the effects of nutrient deprivation on nitrate transport and assimilation, we examined the nitrate accumulation mechanism in the roots and leaves of lettuce. The results revealed that one nitrate transporter protein, *NRT1*, and three *SPX* structural domain DEGs were upregulated in the leaves. In the roots, 16 nitrate transporters were detected, with high-affinity transporters *NRT2.4* and *NRT2.5* being upregulated, indicating that plants adapted to nutrient deprivation by enhancing nitrate assimilation. While, the remaining low-affinity transporters (*NRT1)* were downregulated, suggested that the root absorption capacity is inhibited. Additionally, 10 *SPX domains* were almost all downregulated. One assimilation enzyme, *glutamine synthetase (GS)* was upregulated, which can promote NH_4_^+^ assimilation. While two MADS-box family TFs were downregulated.

Interestingly, under low nitrogen stress, plants typically improve nitrogen absorption efficiency by inhibiting the expression of *bHLH130* (Wang et al., 2022b). However, in this study, *bHLH130* expression was upregulated after nutrient deprivation. These findings suggest that lettuce is assimilated by a unique nitrate redistribution strategy. And a partial list of TFs involved, or potentially involved, in NO_3_^-^ response is provided in **Table 4**.

Therefore, the synergistic regulation of core transcription factors *NRT2, NRT1* and bHLH family member *bHLH130*. These findings systematically clarify the molecular mechanism of nitrogen metabolism reprogramming of lettuce under nutrient deprivation, and provide target genes for the improvement of low nitrate varieties.

### Effects of nutrient deprivation on the antioxidant systems of lettuce

The reactive oxygen species (ROS) scavenging system plays a crucial role in plant defense against abiotic stresses. Under stress conditions, plants enhance their protective mechanisms and survival competitiveness by increasing the production of secondary metabolites (Wang et al., 2022a). Following nutrient deprivation, an imbalance in free radical metabolism within plant cells leads to toxicity and damage, resulting in the accumulation of hydrogen peroxide and superoxide anion (O_2_^-^).

DEGs related to ROS-metabolizing enzymes were identified in lettuce roots. Most *peroxidase (POD)* DEGs were upregulated. *Lipoxygenase (LOX)*, which generates ROS during the catalytic process of unsaturated fatty acids (Zhang et al., 2024c), showed upregulation in 4 out of 5 DEGs. Additionally, two *copper/zinc superoxide dismutase (SODC)* DEGs were downregulated, while most *cytochrome P450* DEGs were upregulated. In the leaves, one *LOX* gene was upregulated, two *SODC* genes were downregulated, and most *cytochrome P450* DEGs were upregulated. These results suggest that DEGs related to *POD, LOX*, and *cytochrome P450* were predominantly upregulated in the roots under nutrient deprivation stress, while *cytochrome P450* and *LOX* were widely upregulated in the leaves, and *SODC*-related DEGs were downregulated in both tissues.

Thus, we infer that the LOX activity in leaves is positively correlated with the POD activity in roots, indicating that roots and leaves adapt resistance through metabolic division of labor: roots are responsible for ROS clearance, and leaves preferentially activate lipid peroxidation signals.

### qRT-PCR validation

**Figure 8:**
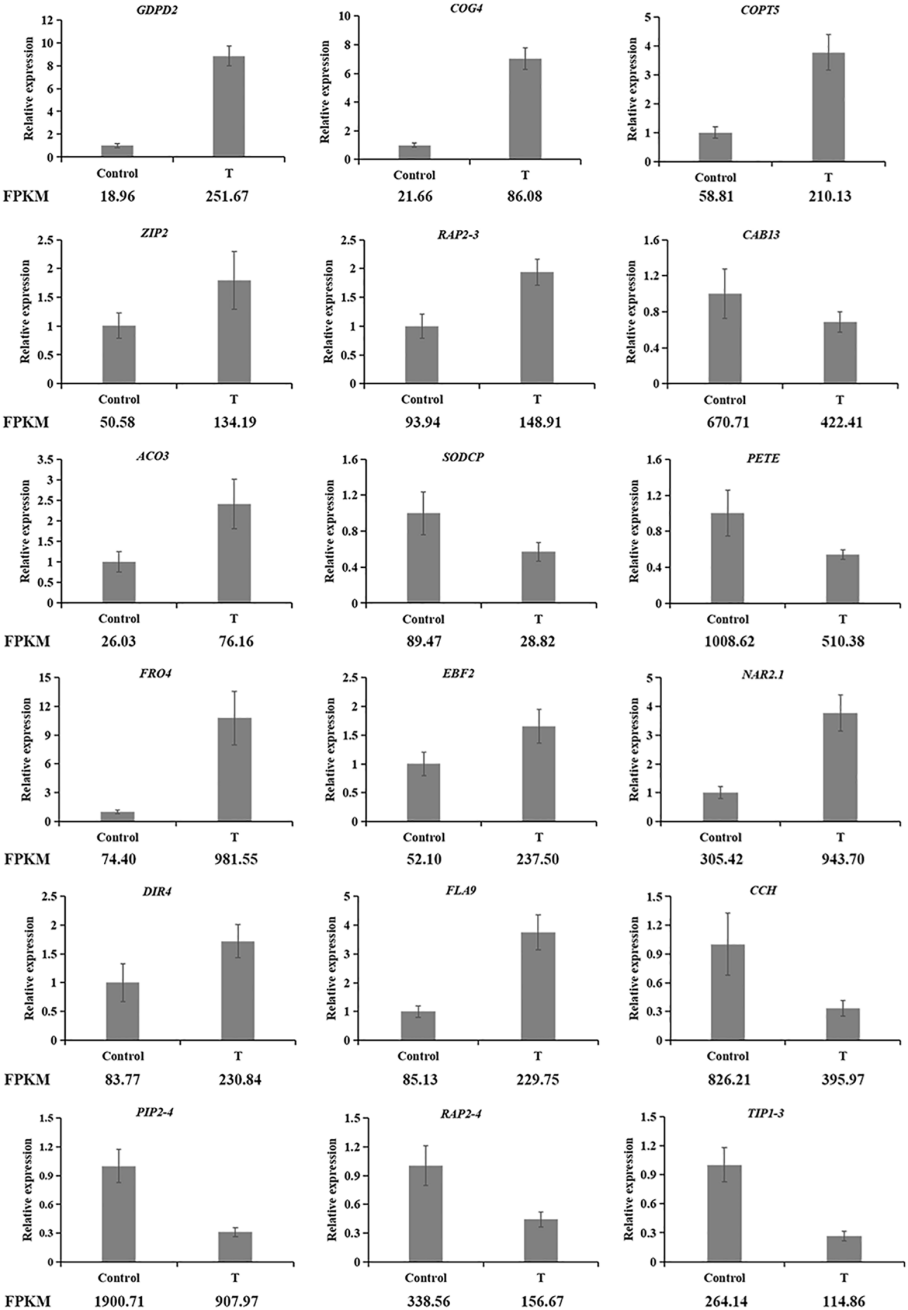
qRT-PCR vertification of DEGs identified by transcriptome analysis (LsACT was used as an internal control. *GDPD2, COG4, COPT5, ZIP2, RAP2-3, CAB13, ACO3, SODCP* and *PETE* are derived from the leaf; *FRO4, EBF2, NAR2.1, DIR4, FLA9, CCH, PIP2-4, RAP2–4* and *TIP1–3* are derived from the root.).

To validate the RNA-seq data, 18 DEGs were randomly selected, and their expression profiles were confirmed via qRT-PCR (**Figure 8**). The results demonstrated that the expression trends observed through qRT-PCR were consistent with those from RNA-seq, confirming the reliability of the transcriptome data. This consistency supports the use of the RNA-seq data for further analysis with confidence.

## Discussion

Nitrogen is a vital element for plant growth and development, with nitrate being the primary form of nitrogen metabolism in vegetables, significantly influencing plant growth and development (Li et al., 2004; Liu et al., 2024a). While, nitrate acted as both a vital nutrient and a potential quality risk factor when accumulated excessively (Okushima et al., 2011). Our study corroborates that nutrient deprivation triggers a transcriptional reprogramming in lettuce, characterized by downregulation of nitrate transporters (e.g., *NRT1.1*) and activation of ammonium assimilation genes (e.g., *GS*). In flue-cured tobacco, midribs with threefold higher nitrate content than lamina exhibited suppressed expression of *NRT2.4* and *NRT2.5*, mirroring our observation that lettuce roots upregulate these high-affinity transporters under nitrogen limitation to enhance nitrate scavenging efficiency. This parallel suggests *NRT2.4/2.5* could serve as universal targets for nitrate reduction across leafy vegetables (Feng et al., 2022; Prasanna et al., 2023).

It has been observed that the nitrate content in vegetables decreases as plant age increases, and appropriate late harvesting can help reduce the nitrate content in vegetables (Liu et al., 2011b; Liu, 2014; Yi et al., 2015). In this study, the nitrate content was determined within 5 days after nutrient deprivation (**Figure 2**), confirming this trend. Additionally, transcriptome analysis of lettuce leaves and roots under normal and nutrient deprivation conditions revealed that upregulated DEGs after nutrient deprivation were primarily involved in cysteine and methionine metabolism, terpenoid backbone biosynthesis, endocytosis, and α-linolenic acid metabolism. The downregulated DEGs were mainly associated with phenylpropanoid biosynthesis, plant hormone signal transduction, carbon metabolism, isoquinoline alkaloid biosynthesis, and purine metabolism (**Figure 6**). These findings align with previous analyses conducted in tobacco and maize (Feng, 2024; Ji, 2024), emphasizing the synergistic regulation of nitrogen metabolism and phenylpropanoid biosynthesis under nutrient deprivation conditions.

Nitrogen deficiency enhances the uptake activity of ammonium and nitrate in plants (Iqbal, 2020). Transcript analysis also revealed significant changes in the expression of genes related to nitrate metabolism in lettuce after nutrient deprivation. These changes likely involve the regulation of nitrate transport, assimilation, and metabolic pathways. For example, the NRT2 family of high-affinity nitrate transporters functions effectively under low nitrate concentrations (Zhang et al., 2024a). Studies in wild soybean have shown that *NRT2.4* and *NRT2.5* are upregulated under low nitrogen stress (Sun et al., 2021; Yu, 2021), and in this study, we found that *NRT2.4* and *NRT2.5* in lettuce roots were similarly upregulated after nutrient deprivation, suggesting these genes play a crucial role in nitrogen absorption and utilization in a low nitrate environment. In the leaves, we found that 1 nitrate transporter protein, *NRT1*, was upregulated after nutrient deprivation. These expression changes may impact the response and metabolism of lettuce under nutrient deprivation stress. *SPX domains* are significantly responsive to low-nitrogen stress. Subcellular localization studies have shown that SPX proteins function in the nucleus and are involved in regulating nitrogen and phosphorus metabolism, as well as plant stress responses (Zhang et al., 2024b). In this study, 3 DEGs with *SPX domains* in leaves were all upregulated. Additionally, the CHLORIDE CHANNEL (CLC) family mediates nitrate absorption from the soil and its translocation and redistribution processes. We detected 1 DEG in both leaves and roots, which further emphasizes the role of CLCs in nitrate transport and distribution in lettuce (Wang et al., 2012, 2018).

In addition, TFs play a crucial role in the regulation of nitrate metabolism. For example, many TFs from the AR2/ERF family are upregulated in lettuce roots and leaves, and WRKY and bZIP family TFs are also involved in plant responses to nitrate stress (Liu et al., 2015; Chen et al., 2017; Jiang et al., 2017; Zhang and Yang, 2017; Pan et al., 2022). In this study, WRKY family TFs (*WRKY18, WRKY60, WRKY70*, etc.) and bZIP family TFs (*bZIP30, bZIP34*) were upregulated in lettuce roots (**Table 4**). The MADS-box family of genes influences growth hormone transport by regulating *PIN* gene expression, thus controlling root growth under varying NO_3_^-^ concentrations (Maurya et al., 2020). In this study, we observed that the expression of two MADS-box family TFs was downregulated after nutrient deprivation. Additionally, the bHLH family gene *bHLH130*, which is significantly downregulated under low nitrogen conditions and negatively correlated with flavonoid pathway genes (Wang et al., 2022b), was upregulated after nutrient deprivation in this experiment. This upregulation of *bHLH130* may suggest a synergistic effect with other TFs to enhance plant tolerance to stress.

Nitrogen deficiency affects plant quality by altering photosynthetic rates, osmotic regulation, and the accumulation of reactive oxygen species, resulting in significant inhibition of growth, development, and appearance (Guo et al., 2023). Wang et al. sequenced the transcriptomes of wheat seedlings treated with low nitrogen and found that the stress response involves signaling, carbon/nitrogen metabolism, and antioxidant activity (Wang et al., 2019). In this study, most of the DEGs related to *POD*, *LOX*, and cytochrome P450 were upregulated in lettuce roots under nutrient deprivation, with most of the *cytochrome P450* and *LOX* genes also upregulated in leaves, while SODC-related DEGs were downregulated in both leaves and roots. These findings suggest that the antioxidant system’s response to nutrient deprivation stress differs between leaves and roots. Additionally, nitrogen deficiency leads to changes in protein expression and enzyme activity, which subsequently alters plant metabolism. For instance, nitrogen deficiency reduces the activity of ribulose bisphosphate carboxylase/oxygenase (Rubisco) (Yang, 2022). In this study, the expression of Rubisco accumulation factor 2 decreased in lettuce roots after nutrient deprivation, indicating potential effects on photosynthesis, respiration, stress tolerance, and secondary metabolite synthesis in lettuce, ultimately altering the plant’s metabolic state.

## Conclusion

This study investigated the effects of nutrient deprivation on nitrate metabolism in lettuce, revealing its impact on growth, development, and metabolism. Nutrient deprivation significantly reduced nitrate content in both lettuce leaves and roots. Transcriptome sequencing analysis indicated that DEGs related to nitrate metabolism were predominantly enriched in pyrimidine metabolism, plant hormone signal transduction, and phenylpropanoid biosynthesis pathways. These findings suggest that lettuce may regulate nitrate metabolism by modulating gene expression in these pathways.

Key nitrate transport and assimilation genes, such as *NRT2.4* and *NRT2.5*, were upregulated in roots, while *NRT1* was upregulated in leaves, indicating an enhanced nitrate transport mechanism under nutrient deprivation stress. Additionally, DEGs related to peroxidase (*POD*), lipoxygenase (*LOX*), and *cytochrome P450* were predominantly upregulated in roots, while *cytochrome P450* and *LOX* were primarily upregulated in leaves. Conversely, DEGs related to superoxide dismutase copper (*SODC*) were downregulated in both tissues.

Nitrogen deficiency also induced changes in protein expression and enzyme activity, affecting plant metabolism. For example, the reduced expression of *Rubisco accumulation factor 2* in roots suggests potential impacts on photosynthesis and overall plant metabolism.

In summary, applying nutrient deprivation 5 days before harvest can effectively reduce nitrate content, enhance nitrogen metabolism, and improve lettuce quality without compromising yield. These results provide valuable insights into the molecular mechanisms underlying lettuce responses to low-nitrogen stress and offer practical guidance for optimizing lettuce cultivation practices.

## Future outlook

This study offers valuable insights into the molecular response of lettuce to nutrient deprivation stress and provides potential molecular strategies for reducing nitrate levels in vegetables. However, additional research is needed to fully elucidate the genetic and molecular mechanisms underlying these responses. Future studies could focus on the functional validation of key TFs identified in this work, as well as investigating the signal transduction pathways and regulatory networks involved in metabolic processes such as nitrate assimilation and oxidative stress response. Furthermore, exploring the role of other potential regulatory molecules and their interactions with environmental factors would be crucial for improving nutrient management efficiency in vegetable production. These efforts will contribute to optimizing strategies for enhancing vegetable quality and reducing harmful nitrate accumulation, ultimately benefiting plant health and consumer safety.

## Data Availability

The RNA-seq raw data used in this study have been deposited in Sequence Read Archive (SRA) database in NCBI under accession number PRJNA1224991 (https://www.ncbi.nlm.nih.gov/sra/PRJNA1224991).
